# Pandemic Dreams: Network Analysis of Dream Content During the COVID-19 Lockdown

**DOI:** 10.3389/fpsyg.2020.573961

**Published:** 2020-10-01

**Authors:** Anu-Katriina Pesonen, Jari Lipsanen, Risto Halonen, Marko Elovainio, Nils Sandman, Juha-Matti Mäkelä, Minea Antila, Deni Béchard, Hanna M. Ollila, Liisa Kuula

**Affiliations:** ^1^Sleepwell Research Program, Faculty of Medicine, University of Helsinki, Helsinki, Finland; ^2^Department of Psychology and Logopedics, Faculty of Medicine, University of Helsinki, Helsinki, Finland; ^3^Department of Psychology and Speech-Language Pathology, University of Turku, Turku, Finland; ^4^Department of Social Psychology, University of Helsinki, Helsinki, Finland; ^5^Visiting Researcher, Institute for Molecular Medicine Finland (FIMM), University of Helsinki, Helsinki, Finland; ^6^Department of Psychiatry and Behavioral Sciences, Stanford University, Palo Alto, CA, United States; ^7^Center for Genomic Medicine, Massachusetts General Hospital, Boston, MA, United States; ^8^Program in Medical and Population Genetics, Broad Institute, Cambridge, MA, United States; ^9^Institute for Molecular Medicine Finland (FIMM), University of Helsinki, Helsinki, Finland

**Keywords:** dream, sleep, crowdsourcing, cluster, network analysis, COVID-19, nightmare, stress

## Abstract

We used crowdsourcing (CS) to examine how COVID-19 lockdown affects the content of dreams and nightmares. The CS took place on the sixth week of the lockdown. Over the course of 1 week, 4,275 respondents (mean age 43, SD = 14 years) assessed their sleep, and 811 reported their dream content. Overall, respondents slept substantially more (54.2%) but reported an average increase of awakenings (28.6%) and nightmares (26%) from the pre-pandemic situation. We transcribed the content of the dreams into word lists and performed unsupervised computational network and cluster analysis of word associations, which suggested 33 dream clusters including 20 bad dream clusters, of which 55% were pandemic-specific (e.g., Disease Management, Disregard of Distancing, Elderly in Trouble). The dream-association networks were more accentuated for those who reported an increase in perceived stress. This CS survey on dream-association networks and pandemic stress introduces novel, collectively shared COVID-19 bad dream contents.

## Introduction

On March 11, 2020, the World Health Organization announced that COVID-19 is a pandemic (World Health Organization, 2020). Significant events and threatening situations change the way people sleep and dream, and pandemics are no exception (Nielsen et al., 2006; Hartmann and Brezler, 2008; Sandman et al., 2013; Cenat et al., 2020). While sporadic changes in sleep and dreaming are normal, and sleep naturally responds to environmental fluctuation, extreme factors and traumatic experiences can lead to severe changes in sleep patterns, including altered dream content or more nightmares. An increase in nightmares has been previously observed with regard to wars, terrorist attacks, and during earlier pandemics or infectious diseases (Nielsen et al., 2006; Hartmann and Brezler, 2008; Sandman et al., 2013). Similarly, the COVID-19 pandemic poses a threat to health and well-being, causes worries and fears, alters behavior and everyday life, and has a lasting impact on how we perceive the world (Zhou et al., 2020). Thus far, preliminary reports suggest that poor-quality sleep and insomnia-related symptoms have indeed increased in the general population (Casagrande et al., 2020; Huang and Zhao, 2020; Xiao et al., 2020); however, the impact of the pandemic on the content of dreams and nightmares remains unexplored. Seizing on the opportunity and collecting dream content during a carefully selected time window during the COVID-19 lockdown allowed for a unique natural experiment to study dreams and nightmares.

Indeed, dreams reflect waking consciousness by spontaneously incorporating daytime experience (Koulack et al., 1985; Stickgold et al., 2000; Nielsen et al., 2004; Malinowski and Horton, 2014; Solomonova et al., 2015). Evidence converges to suggest that the incorporation of experience into dreams is characterized by a *circaseptan* process, such that dreams include events from both the preceding day (*day-residue effect*) and a week earlier (*dream−lag effect*) (Nielsen et al., 2004). In order to understand whether the stress of the pandemic influences dreams and results in collectively shared dream content (i.e., whether shared experiences, such as COVID-19 and the lockdown, produce similar dreams), it is crucial to solicit the dream reports during a very short timeframe.

Nightmares are a special case of dreaming. They can be defined as repeated occurrences of extended, extremely dysphoric, and well-remembered dreams that usually involve threats to survival, security, or physical integrity (American Psychiatric Association, 2013). Current definitions acknowledge that many nightmares are not followed by an awakening (American Psychiatric Association, 2013). While occasional nightmares are common and harmless, frequent nightmares have been associated with other sleep problems (Zadra and Donderi, 2000), symptoms of depression (Li et al., 2010), and even increased risk of suicide (Russell et al., 2019). Idiopathic nightmares can contain events and themes that reflect daytime experiences, but they are rarely repetitive or replicate events accurately (Gieselmann et al., 2019). Post-traumatic nightmares, on the other hand, have a clear relation to previously experienced traumatic events and may even replicate parts of the trauma in detail (Sandman et al., 2013; Miller et al., 2017). The COVID-19 pandemic and the lockdown had potential to generate both types of nightmares, depending on the circumstances of the dreamer.

The current study used a crowdsourcing (CS) method to investigate how COVID-19 lockdown is associated with self-reported changes in perceived stress and increases in nightmares or deviations in dream content. We took the opportunity to ask respondents to describe the content of their dreams during lockdown and then applied computational network and cluster analyses to find clustered word-association chains commonly occurring in the data. In keeping with the dream continuity hypothesis (Schredl and Hofmann, 2003), we expected dreams to exhibit novel thematic categories specifically related to the current pandemic lockdown.

## Results

### Sample Characteristics

There were 4,275 citizens who participated in the CS (mean age 42.6, SD = 13.7 years, range 10–99, 79% women, 19% men, 2% other/unspecified) over the course of 1 week, and 811 respondents reported their dream content. The age distribution of the participants is shown in Table 1. The majority (57.5%) were employed and worked from home, and the remainder had the following distribution: 12% working outside the home; 10.2% students; 8.6% retired; 8.7% unemployed/laid off; and 3.2% other. Of the participants, 24.8% lived in a single-person household, 35.3% lived with their spouse, 34.5% lived in a household with children, 2.8% lived in a shared household, and 2.6% did not specify.

**TABLE 1:** Age distribution of the sample.

Years	*N*	%
<20	75	2.2
20–34	1051	30.1
35–49	1273	36.5
50–64	824	23.6
≥65	264	7.6

*788 respondents did not report age.*

### Perceived Stress and Sleep During the Lockdown

Perceived stress levels increased in the majority (56%) of respondents [high increase in 767 (18.0%), whereas 1,620 (38.0%) reported a modest increase]. A total of 933 (21.9%) respondents reported unchanged, and 943 (22.1%) reported lowered stress. Stress levels increased in more females than males (*P* < 0.001). The pandemic’s impact on sleep patterns was assessed in terms of sleep duration, sleep latency, awakenings, the regularity of the sleep rhythm, and the frequency of nightmares (Table 2). Females were more likely than males to report increased sleep duration and more frequent nightmares (*P* < 0.001 and *P* < 0.001, respectively).

**TABLE 2:** Change in sleep patterns from pre-COVID-19 to lockdown.

	Sleep duration	Sleep latency	Awakenings	Sleep regularity	Nightmares
Decreased (%)	16.6	13.0	11.7	16.3	3.5
No change (%)	29.1	77.4	59.7	53.8	70.4
Increased (%)	54.2	9.6	28.6	29.9	26.0

**Lockdown-associated sleep changes per respondent**					

Number of sleep items	1	2	3	4	5
Complaints (%)	24.0	15.6	8.7	3.1	0.6
Improvements (%)	32.1	20.8	8.7	2.6	0.4

**FIGURE 1:**
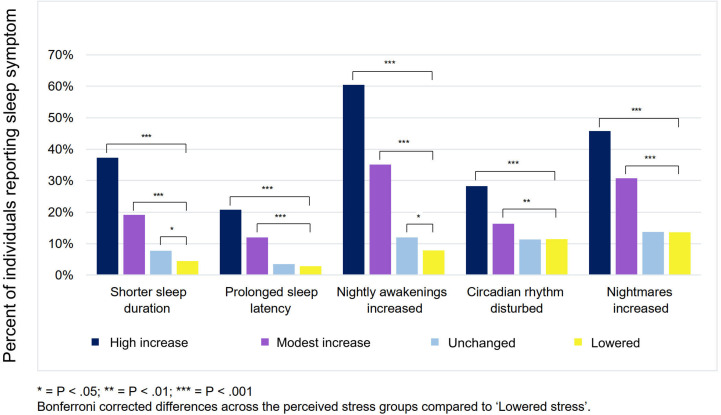
The percentage of individuals reporting worsening sleep (shorter sleep duration, longer sleep latency, more awakenings, disturbed circadian rhythm, and more nightmares) according to their experience of COVID-19–related stress.

An increase in perceived stress levels was associated with progressively shorter sleep duration (*r*_s_ = 0.26, *P* < 0.001), prolonged sleep latency (*r*_s_ = 0.37, *P* < 0.001), more frequent nightly awakenings (*r*_s_ = 0.43, *P* < 0.001), increasingly irregular sleep rhythms (*r*_s_ = 0.182, *P* < 0.001), and more frequent nightmares (*r*_s_ = 0.29, *P* < 0.001). Figure 1 displays the prevalence of sleep disturbances in relation to respondents’ perceived levels of stress. Increased disturbances were concentrated primarily in those who reported a high increase in stress; their sleep worsened linearly with the increase in perceived stress. The most prominent increases were for nightly awakenings (60.5%) and nightmares (45.8%) in the groups with the highest perceived stress (*P* < 0.001).

Regarding beneficial effects, those with lowered stress levels during the lockdown reported longer sleep duration (73.9%) and more regular sleep rhythm (44.9%) compared to their pre-pandemic sleep.

### Dream Content Frequencies and Networks

Dreams were reported by different frequencies in different stress groups (high increase in stress = 27.4.5%; modest increase in stress = 20.5%; stress unchanged = 13.0%; stress lowered = 17.8%), χ^2^ = 58.427, *P* < 0.001. Age was not associated with the likelihood of reporting dreams (*P* = 0.087). Females were more likely to report dream content than males (χ^2^ = 72.423, *P* < 0.001; 21.6 and 9.6%, respectively). In addition, working status differed between dream reporters and non-reporters (χ^2^ = 23.022, *P* < 0.001). Students and retired persons were more likely to deliver a dream report, whereas those working remotely were less likely (Supplementary Figure S1).

**FIGURE 2:**
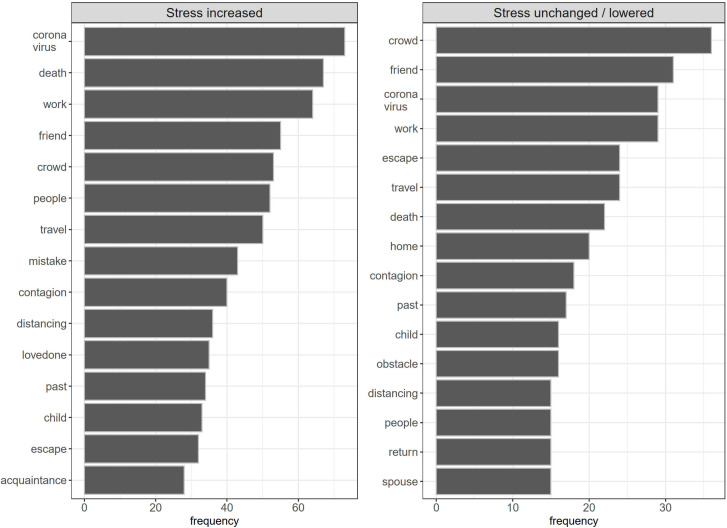
The appearance frequency of different words in dream reports according to the experience of COVID-19–related stress (left panel: stress increased; right panel: stress unchanged/lowered).

Figure 2 displays the most frequent words in the dream report data: column 1 for “stress increased” (words: *coronavirus*, *death*, *work*; modest or high increase responses combined) and column 2 for “Stress-unchanged/lowered” (words: *crowd*, *friend*, *coronavirus*).

Next, we created an unsupervised network analysis in which word associations are displayed in a correlation network. Unsupervised cluster analysis suggested 33 dream clusters in all of the groups combined, 27 in the “stress increased” group, and 11 in the “stress unchanged/lowered” group. Only words with a minimum of five occurrences and correlations >0.2 were retained in the final network models.

**FIGURE 3:**
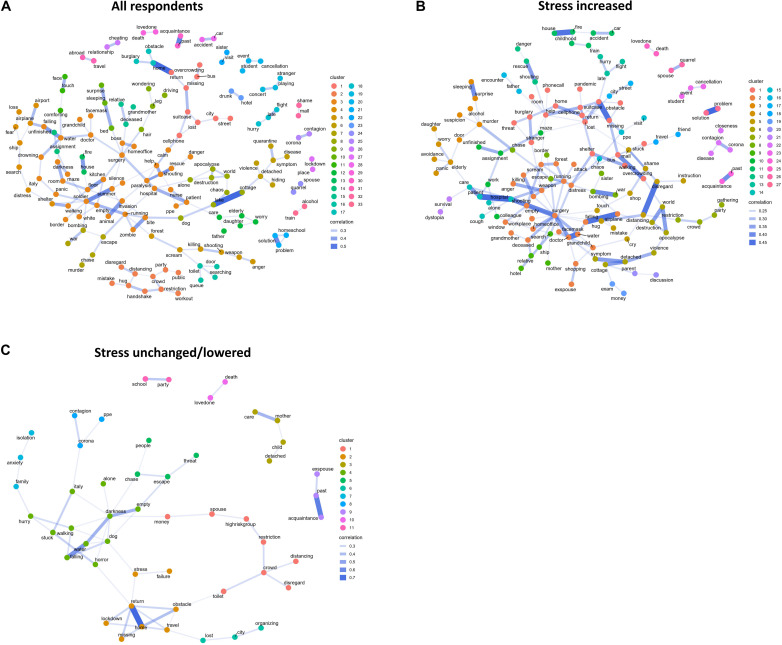
Dream content clusters according to perceived stress in panel **(A)** all respondents, panel **(B)** in respondents with stress increased, panel **(C)** in respondents with stress unchanged/lowered. Colors refer to computational clusters, where dots/words with similar color belong to the same cluster. The level of strength of association between two words is illustrated with a stronger line between them. Lines indicate a statistically significant association.

Figure 3 displays the dream clusters in groups based on stress level. After performing exploratory computation, we qualitatively analyzed the content of each dream cluster and labeled it accordingly (Table 3).

**TABLE 3:** Dream cluster labels according to perceived stress.

All respondents	Stress increased	Stress unchanged/lowered
1. *Travel Difficulties, overcrowding 2. *Disregard of distancing 3. *Surgery and troubles 4. Travel 5. *Violence with weapons 6. *Quarantine and disease symptoms 7. *War 8. *Apocalypse 9. Body and hair **10. Touch** 11. Nightly surprise 12. *Elderly in trouble 13. *Fire 14. *Unfinished task 15. Deceased grandmother 16. *Toilet and queuing 17. *Burglary 18. *Being late 19. Concert 20. *Event cancellation **21. Homeschool** 22. Sister visit 23. Being drunk 24. *Quarrel with spouse 25. *Coronavirus contagion 26. Cheating in relationship 27. *Lockdown 28. *Car accident 29. *Death of loved one 30. People from past 31. Travel abroad 32. *Shame in public place 33. Drinking alcohol **11/20 of mainly distressing dream clusters are pandemic-specific (55%)**	1. *Travel Difficulties, overcrowding 2. *Falling 3. *Murder in the night 4. *Elderly in trouble 5. *Disregard of distancing 6. *Apocalypse 7. *Crowd-restriction 8. *Chase-escape in war and borders 9. *Searching the lost 10. *Unfinished task 11. *Transport accident 12. *Being in danger and shouting 13. *Being late 14. *Patient care, cough 15. *Personal protective equipment 16. Father 17. City landscape 18. Travel 19. *Exam 20. Discussion with parent 21. *Dystopia 22. *Coronavirus contagion 23. *Event cancellation 24. People from past 25. *Death of loved one 26. *Quarrel with spouse 27. Problem solution **11/21 of mainly distressing dream clusters are pandemic-specific (52%)**	1. *Disregard of distancing 2. *Obstacles in returning home 3. Care of family 4. *Falling into dark water 5. *Chase-escape 6. *Being lost in a city 7. *Isolation anxiety 8. *Coronavirus contagion 9. People from past 10. *Death of loved one 11. School party **4/9 of mainly distressing dream clusters are pandemic-specific (44%)**

Bolded labels rated as pandemic-specific. *Bad dreams or nightmares.

A subset of the dreams were highly pandemic-specific. For example, cluster 2 (all respondents) comprises three-dimensional associations between *mistake–hug*, *hug–handshake*, *handshake–restriction*, *handshake–distancing*, *distancing–disregard*, *distancing–crowd*, *crowd–restriction*, and *crowd–party*, and we labeled it “Disregard of Distancing” and rated it as pandemic-specific. As another example, cluster 1 (all respondents) included the following word associations: *return–missing*, *missing–suitcase*, *missing–bus*, *bus–overcrowding*, *suitcase–lost*, *lost–city*, *city–street*. While some of the clustered words referred to idiopathic nightmare content, the association with overcrowding made it pandemic-specific. We labeled it “Travel Difficulties, Overcrowding” (Table 3). As an example of overlapping clusters, cluster 1 overlapped with cluster 17 (“Burglary”) through the word *return* in both clusters (*return–home*, *home–burglary*, *home–obstacle*).

The number and content of identified clusters varied according to the stress level. While most of the clusters’ contents related to themes that could be clearly identified through their association networks, a few of the COVID-19–related clusters included large association networks of different pandemic-associated threats and landscapes.

**FIGURE 4:**
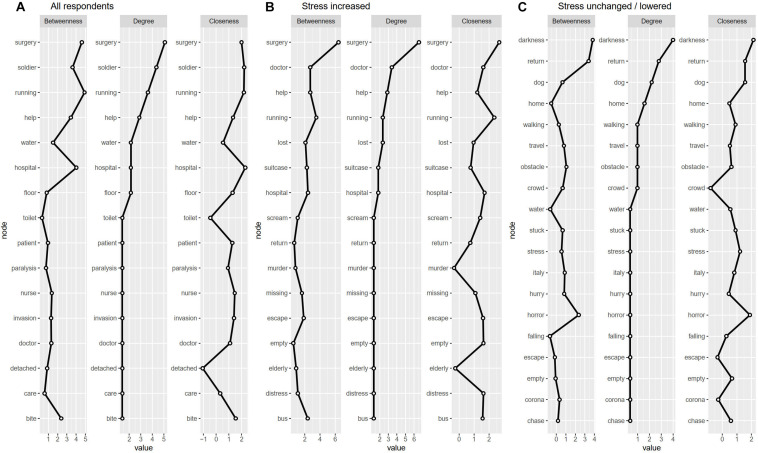
Network analysis parameters according to the perceived stress in panel **(A)** all respondents, panel **(B)** in respondents with stress increased, panel **(C)** in respondents with stress unchanged/lowered. Betweenness is measured as the number of times word X lies on the shortest path between words Y and Z. Degree centrality is simply the sum of direct (i.e., adjacent) edges that each word has with other words. Closeness measures how close a word is to other words and is inversely proportional to the mean shortest distance to all other words.

Figure 4 shows that the data resulted in the following words that were most central, i.e., most frequently connected with other words independently of each other (degree of centrality): *surgery*, *soldier*, *running* (in the group of all respondents); *surgery*, *doctor*, *help* (in the group where stress was increased); and *darkness*, *return*, *dog* (in the group where stress was unchanged/lowered). The three most frequent node words in terms of betweenness (i.e., the number of times word X lies on the shortest path between words Y and Z) were *surgery*, *running*, *hospital* (in the group of all respondents), *surgery*, *running*, *bus* (stress increased group), and *darkness*, *return*, *horror* (stress unchanged/lowered group). The three most frequent words in terms of closeness indicators were *surgery*, *running*, *hospital* (in the group of all respondents); *surgery*, *running*, *hospital* (stress increased group); and *darkness*, *horror*, *return* (stress unchanged/lowered group). The “stress increased” group had pandemic-specific network nodes, whereas nodes in the “stress unchanged/lowered” group reflected conventional nightmare/bad dream content.

## Discussion

We explored sleep patterns and pandemic dream content during the sixth week of the lockdown. Instead of traditional *a priori*–defined thematic content analysis of dreams, we used a computational exploratory, unsupervised network analysis in which single words were associated to create a network of dream content (Pons and Latapy, 2005). These networks were clustered based on the association networks around single words. The resulting dream clusters allowed identification of novel and commonly shared dream content during a time-restricted lockdown period.

The impact of lockdown on sleep quality was twofold. The majority of the respondents reported having a longer sleep duration, and almost a third reported having more regular sleep rhythms during the lockdown compared to the pre-lockdown situation, likely reflecting alleviated pressure in scheduling due to working from home. Lockdown-related sleep complaints were also common: almost a third reported more frequent awakenings at night, and more than a quarter of the respondents also had an increase in nightmares. As could be expected (Nielsen et al., 2006; Nielsen and Levin, 2007; Robert and Zadra, 2014), we observed a larger increase in the frequency of nightmares among respondents who also reported elevated stress during the lockdown, although no causality can be inferred.

Our analysis of dream content revealed typical, idiopathic nightmares/bad dreams (e.g., falling, being chased, being late, and the deaths of loved ones) (Robert and Zadra, 2014), comparable to nightmares reported in a very similarly aged population-representative sample in 2010 (mean age = 46, SD = 16 years) (Schredl, 2010). Health-related concerns represented 12% of the distressing dream content in the general population (Robert and Zadra, 2014). In the current study, we identified 33 different dream clusters (in all respondents), of which 20 were evaluated as having distressing dream content, and of these, 55% were pandemic-specific. Themes such as failures in social distancing, coronavirus contagion, personal protective equipment (PPE), dystopia, and apocalypse were rated as pandemic-specific. Most of these were confounded with established idiopathic dream categories (e.g., failure, death, worry), but the dream imagery was specific to the current pandemic situation.

Sleep enables offline processing of recent memory traces (Peigneux et al., 2003). Sleeping enhances newly acquired memories through a physiological reactivation of brain areas recruited during learning (Klinzing et al., 2019), as activation in these specific brain regions is known to correlate with particular dream content (Siclari et al., 2017). Fear-related dream imagery (often experienced in bad dreams, idiopathic nightmares, and post-traumatic nightmares) associated with emotional arousal can equally serve to extinction of fear memories (Nielsen and Levin, 2007) and would then assist in the emotional adaptation to the presence of COVID-19, as REM sleep plays a critical role in emotional processing (Tempesta et al., 2018). Dreams related to failures in social distancing, for example, may then help in consolidating episodic memory for new behavioral rules and routines in social situations.

Hartmann’s contextualizing hypothesis proposes that nightmares serve the function of contextualizing, or finding a picture context for, an individual’s predominant emotional concerns (Hartmann, 1998; Hartmann and Brezler, 2008). In line with that, our analytical approach to associative dream content networks was particularly insightful in terms of revealing pandemic-specific dream imagery, as we detected some central dream images (e.g., surgery, PPE) that may refer to fear but were contextualized in varying dream events. Overall the findings from analysis of dream content are consistent with both *threat simulation theory* (Revonsuo, 2000) and the *dream continuity hypothesis* (Schredl and Hofmann, 2003), as we identified dreams that potentially prepare the dreamer for negative events that may take place (threat simulation) and furthermore replicate those events that are observed during waking (continuity).

Comparing the dream-association networks according to perceived (daytime) stress, we found fewer repeated association networks in the “stress unchanged/lowered” group, but roughly half of the dream-association networks were still pandemic-specific, as was also the case in the “stress increased” group. The most frequent dream words were *corona[virus]*, *crowd*, and *death* among those with increased stress levels, and *crowd*, *friend*, and *corona[virus]* among those with unchanged/lowered stress levels. This would suggest that the pandemic has a substantial impact on the content of dreams independently of perceived stress. However, the available information could not distinguish between post-traumatic nightmares and the incorporation of waking events and worries into the dream context without related emotion.

A particular strength of the study is in computational analysis of the dream reports. The analyses of dream reports with unsupervised algorithms were then free from *a priori* defined dream categories. As another asset of this study, the CS was open only during 1 week, allowing investigation of time-bound dream content in relation to the progress of the COVID-19 lockdown. Just 10 days after the CS ended, the COVID-19 restrictions were partly lifted (for example, schools were reopened). The time-bound nature of the CS is important as dreaming is thought to constitute a valuable model for the study of consciousness with implications beyond sleep (Siclari et al., 2017). This is because in normal sleep we are at least partly disconnected from environmental input and from performance of any tasks. The current approach may also be inspected from the perspective of dreams as a collectively shared consciousness during the COVID-19 lockdown.

In the same vein, these interpretations are also limited by a number of methodological constraints. Dream reports are never fully accurate, and they are confounded by a number of factors: elapsed time, memories, other dreams, forgetting, and translation to a verbal narrative (Waterman et al., 1993; Windt, 2010). Some people recall very specific and visually rich dreams, whereas the dream reports of others contain only one specific dream event, such as being chased. The newspaper article on stress and nightmares published jointly with the invitation to the CS questionnaire may also, apart from inspiring respondents to report their dreams, cause systemic bias. In addition, our classification of nightmares/bad dreams was not based on subject ratings, as is typically the case; rather, our panel of judges made qualitative judgments as to whether or not dreams were distressing. The CS might have attracted more responders with sleep problems or with a fresh experience of a pandemic-specific dream, resulting in a systemic bias. We also acknowledge that the data analytic steps included human-made decisions, such as harmonizing words and expressions, and classification of dream report content as pandemic or non-pandemic. While the method we applied is completely novel in dream report analysis, it required intensive teamwork and shared decision-making processes. This study did not then allow for the testing of inter-rater reliability. Additionally, the groups with different stress levels were not even in terms of dream report material, as participants with increased stress reported more frequently dreams, and consequently, the data and the related association networks were wider. It is of note that while some cluster networks, especially in the low stress group, were very narrow and easy to label, some cluster networks were wide and partly unfocused. Labeling clusters is based on qualitative interpretations and coming to a consensus on the best fitting label. Yet, the process is fully transparent as all cluster data are openly presented in the Figure 3.

To sum up, we applied a network analysis to explore dream content during COVID-19 lockdown. We found several pandemic-specific dream contents and dream imagery that were associated with a variety of distressing events. A large proportion of the respondents had increased stress, which was associated with nightmares and sleep disturbances. Because sleep disturbances and nightmares are known to predict depression and a range of other mental health problems (Li et al., 2010; Sandman et al., 2015; Baglioni et al., 2016), a consequence of the COVID-19 pandemic may be that mental health is being impaired around the world.

## Materials and Methods

### Crowdsourcing

**FIGURE 5:**
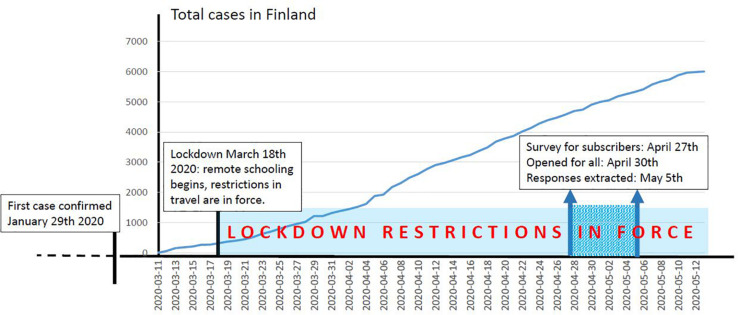
Timing of the crowdsourcing survey from the first confirmed COVID-19 case in Finland to May 5, 2020, when the current data were extracted. Light blue indicates the period under lockdown restrictions, including remote schooling and travel restrictions within Finland and abroad. In Finland, individuals were allowed to leave their house freely and spend time outdoors. Gathering size was limited to 10. There were no restrictions relating to outdoor activities, and public transport was available. A period in darker turquoise marks the timing of the data collection.

Figure 5 presents the timeline of the pandemic onset and lockdown in Finland, as well as the timing of our CS survey on sleep. We collaborated with the most widely circulated newspaper in Finland [*Helsingin Sanomat* (HS), weekly reach of readers *N* = 2 M, average daily readers *N* = 339,437] to generate broad participation in the very short timeframe of 1 week. The short time window maximized the situational context, which was particularly important for the dream reports. The survey was launched attached to a digital newspaper article on sleep (published April 27, 2020). On January 5, 2020, HS published a reminder that the survey is ongoing and now without paywall. The data were extracted on May 5, 2020, for the analyses. All readers were invited to respond through an internet survey, which gathered data regarding sleep during the COVID-19 lockdown. By filling in the questionnaire, the respondents also consented to the anonymized data being delivered to the Sleep and Mind Research Group at the University of Helsinki, Finland. The consent formulation was as follows: “By filling in the questionnaire, you consent to report your sleep and dreaming to *Helsingin Sanomat* (newspaper) and to researchers about how the pandemic lockdown has influenced your sleep. Please leave your name and contact information if your responses can be directly cited in the newspaper article. Your name will not be published, but *Helsingin Sanomat* requires the identity of the respondents to be known by the newspaper if they are directly cited. The newspaper office will not distribute your personal information to third parties. Responses to the questionnaire may also be used by the Sleep and Mind Research Group at the University of Helsinki. *Helsingin Sanomat* will only share anonymized data (without name and contact information) with the Sleep and Mind Research Group.” We asked the respondents to assess how their sleep patterns had changed since the COVID-19 lockdown with regard to sleep duration, number of awakenings, sleep latency, frequency of nightmares (*increased*, *not changed*, *decreased*), and sleep rhythm regularity (*change/no change*). An evaluation of perceived stress was also grounded in how they believed their levels of stress had changed since the lockdown began (*high increase*, *modest increase*, *unchanged*, *lowered*, *that were further aggregated to two groups*: *stress increased and stress unchanged/lowered*).

### Treatment of the Dream Content

The respondents were asked the following question: “Describe your dreams during the pandemic lockdown” (which included open space to write). Some respondents wrote two dreams, which were treated as separate reports. While mainstream studies of nightmares and distressing dreams have used *a priori* defined thematic grids in the dream content classification (Robert and Zadra, 2014), the current study was purely computational, without any predefined dream-content grids. This method allowed an exploratory analysis of the contents of dreams that has not been used before in dream research. However, before the computational phase, the data were harmonized; for instance, dream reports were reduced to word lists containing the essential nouns in the dream report. The dream reports were first transcribed into chronological word lists for each dream separately. For example, a dream report (“I had tuberculosis and the doctor was angry with me because I did not come to see him when the first symptom appeared”) was transcribed to “tuberculosis-doctor-anger-symptom.” After the first draft of the transcripts was created, synonymous words and the spelling format of different words (e.g., *shoe vs. shoes*) were harmonized. If a word appeared twice in the dream script, it also appeared twice in the harmonized version to maintain the chronological order of the events. In addition to harmonizing the dream scripts according to the nouns, harmonizing also involved grouping some word types into categories. For example, all relatives beyond father, mother, grandfather, grandmother, daughter, and son (that were retained as such) were grouped under “relative.” Recall of specific cities was grouped under “city,” whereas different country names were retained. Synonymous words or expressions were replaced with one chosen word. For example, there were many different expressions for “overcrowding,” which was the word that we chose in the harmonization process. The original reports were in Finnish, and translation into English required harmonization, as the vocabulary in different languages differs (Finnish: *paljon ihmisiä [a lot of people]*; English: *crowd*). Compound words in Finnish were retained as compound words in English, even when the equivalent was not linguistically correct (e.g., *high-risk-group*; *loved-one*). References to coronavirus were retained as *corona*, as in the original language. All dream scripts were reviewed at least twice, and all harmonization decisions were done collectively after discussion between five experts. The harmonized dream reports included 4,743 words, out of which 1,095 were unique (the respective numbers were 1,514 and 589 among stress unchanged/lowered and 3,226 and 877 among stress increased group). Note that the Finnish language is morphologically highly complex, and harmonization could not be done computationally, as has been done in English text analytics (Kettunen, 2006). We did not ask whether the respondents experienced their dream as distressing/bad, but the panel classified the dreams based on their content as bad dreams (or not) based on a shared panel view. If the resulting dream word clusters included clearly lockdown or pandemic-specific word associations, the panel judged the dream as pandemic-specific. The assessment was fully qualitative, and to ensure full transparency of the classifications, we show the word association source data and the clusters fully in Figure 3.

### Statistical Analyses

We used Bonferroni-corrected one-way analysis of variance in examining sex differences in stress and sleep measures. Spearman correlations were used to study how perceived stress level was associated with self-reported sleep. Pearson χ^2^ was used to test if the relative frequency of reporting a dream differed between age, sex, and demographic status. The associations between perceived stress level and dream reports were analyzed in the following steps: (1) we divided the respondents according to their perceived stress level into “stress increased” and “stress unchanged/lowered;” (2) we calculated the most frequently used words in dream descriptions in both groups; (3) we analyzed the associations between words by tokenizing each dream with pairs of adjacent words (dividing each dream description into word pairs); (4) we calculated the frequency of words co-appearing and the pairwise phi correlations/distances between words (Silge and Robinson, 2016). Phi correlation is a common measure for binary correlation. The focus of the phi coefficient is how much more likely it is that either both words X and Y appear, or neither does, than that one appears without the other. In the final step (5), we illustrated these associations separately for correlations of co-appearance, creating networks for the groups with “increased stress” and “stress unchanged/lowered,” and estimated both clustering (using Spinglass algorithm) of the words and degree centrality (strength) (Pons and Latapy, 2005). Spinglass algorithm is a community detection algorithm that finds groups of words that are closely connected to each other and tend to co-occur (Reichardt and Bornholdt, 2006). Of the different algorithms, it is particularly suitable for the size of the current dataset (Yang et al., 2016).

The word association networks were also analyzed using statistical indexes. Centrality represents the connectedness of a given node (words) with all the others in the network. Node-strength centrality was defined as the sum of all associations that a given node (word) exhibits with all others in the network (the sum of the weighted number and strength of all connections of a specific node relative to all other nodes) (Freeman, 1978; Valente and Foreman, 1998). Betweenness and closeness measures are based on the shortest path length (number of connections form node to another) connecting any two nodes (words). A word with high betweenness lies along the shortest path connecting many other words in the network. Thus, the target word would have a high betweenness centrality if it appears in many shortest paths (Freeman, 1977). Closeness is calculated as the reciprocal of the sum of the length of the shortest paths between the node and all other nodes in a network; it is useful for finding the words that are best placed to influence the entire network most quickly. Thus, the more central a node is, the closer it is to all other nodes (Sabidussi, 1966). These analyses were conducted using R 3.6.1. (Pedersen, 2017).

## Data Availability Statement

The datasets presented in this article are not readily available because the crowdsourcing was performed by *Helsingin Sanomat* who owns the data. Requests to access the datasets should be directed to anukatriina.pesonen@helsinki.fi.

## Ethics Statement

Ethical review and approval was not required for the study on human participants in accordance with the local legislation and institutional requirements. The patients/participants provided their written informed consent to participate in this study.

## Author Contributions

A-KP as the PI of the study designed the study and data collection, led the dream data preparation, and manuscript writing. JL designed and conducted the network analyses and contributed to manuscript writing. RH and ME prepared the dream data, assisted in statistical analyses, and contributed to manuscript writing. NS contributed to manuscript writing and critically reviewed it. JM-M and MA prepared the dream data and critically reviewed the manuscript. DB critically reviewed the manuscript. HO contributed essentially to the manuscript writing. LK contributed essentially to each stage of the data collection and manuscript writing. All authors contributed to the article and approved the submitted version.

## Conflict of Interest

The authors declare that the research was conducted in the absence of any commercial or financial relationships that could be construed as a potential conflict of interest.

## Supplementary Material

Supplementary Figure 1 Percentage of respondents delivering a dream report according to their working status.
