# Supplementary figures and images for: Pandemic Dreams: Network Analysis of Dream Content During the COVID-19 Lockdown
Source: Front Psychol. 2020 Oct 1;11:573961. doi: 10.3389/fpsyg.2020.573961 (PMC7560506; doi:10.3389/fpsyg.2020.573961)

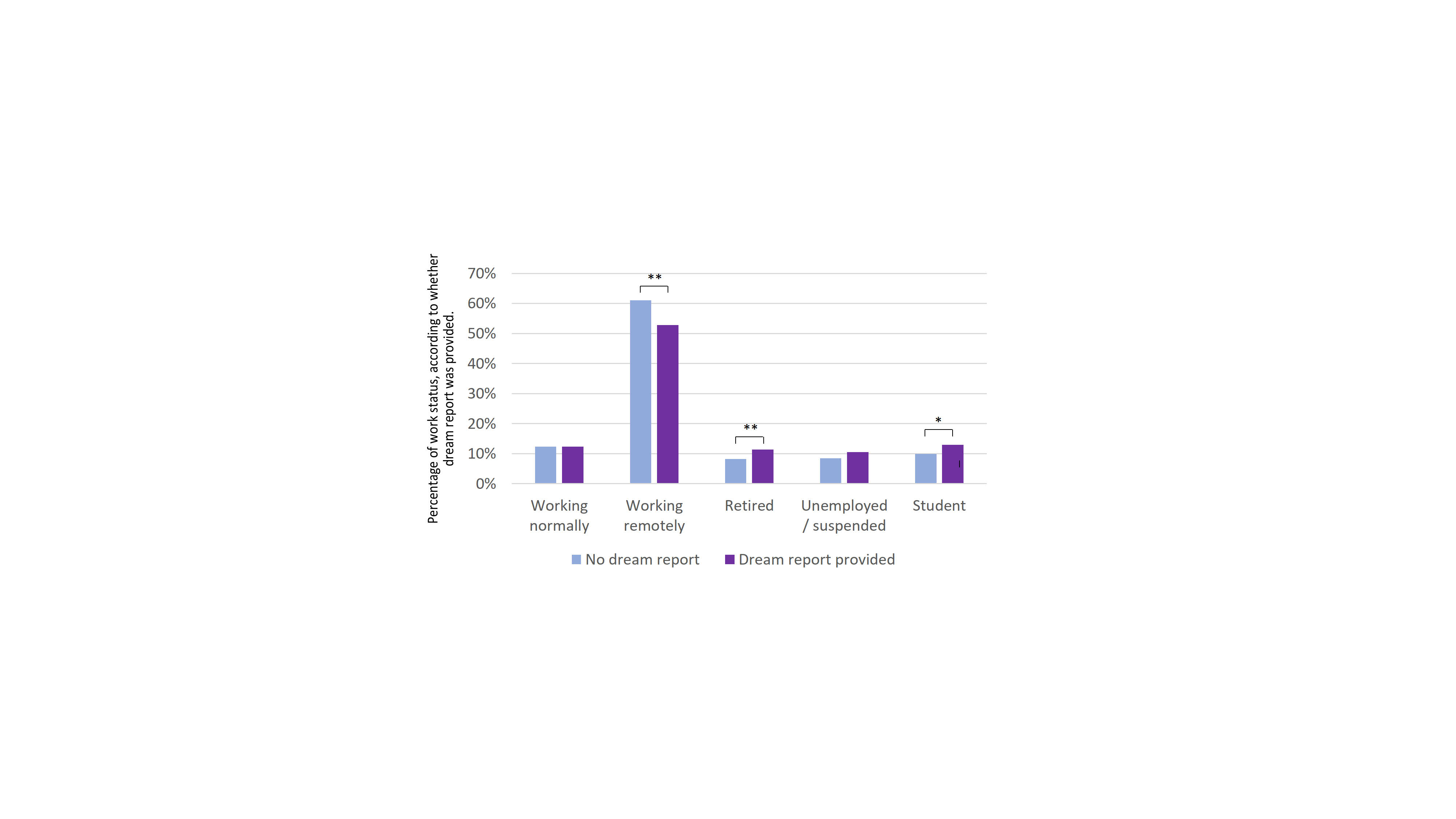

Supplement: Supplementary Figure 1 — Percentage of respondents delivering a dream report according to their working status. [file Image_1.JPEG]
